# Recurrent Guillain–Barré Syndrome Following Ischemic Stroke: A Rare Case With a Four-Year Inter-Episode Interval

**DOI:** 10.7759/cureus.96347

**Published:** 2025-11-07

**Authors:** Binyam M Habte, Yoseph M Habte, Biruk Woldeyohanes, Biruk T Wubneh, Esimael M Abdu

**Affiliations:** 1 Department of Medicine, ALERT Comprehensive Specialized Hospital, Addis Ababa, ETH; 2 Department of Medicine, Ethio Tebib Hospital, Addis Ababa, ETH; 3 Department of Medicine, Axon Stroke and Spine Centre, Addis Ababa, ETH; 4 Department of Medicine, University of Gondar, Gondar, ETH; 5 Department of Surgery, Teklehaimanot General Hospital, Addis Ababa, ETH

**Keywords:** case report, guillain- barré syndrome, post-stroke neuromuscular complications, recurrent guillain-barré syndrome, stroke-associated guillain–barré syndrome

## Abstract

Guillain-Barré syndrome (GBS) is an acute immune-mediated polyradiculoneuropathy that typically follows infections and is usually monophasic, with recurrence occurring in only a few cases. Its development after cerebrovascular events is exceedingly rare, complicating timely recognition due to overlapping neurological deficits. We describe a 67-year-old man who, four years earlier, experienced an acute right basal ganglia ischemic stroke and, on the fourth day of hospitalization, developed rapidly progressive quadriparesis and areflexia. Initial suspicion for stroke recurrence or hemorrhagic transformation was excluded by neuroimaging. Cerebrospinal fluid analysis revealed albuminocytologic dissociation, and nerve conduction studies demonstrated acute motor-sensory axonal neuropathy (AMSAN), confirming GBS. He was treated with intravenous immunoglobulin, achieving a gradual recovery. Four years later, he presented with spontaneous ascending weakness and respiratory compromise. Cerebrospinal fluid protein was elevated, and repeat nerve conduction studies showed acute inflammatory demyelinating polyneuropathy (AIDP). Plasmapheresis, supportive care, and structured rehabilitation resulted in substantial neurological improvement. This case highlights the rare occurrence of GBS following stroke and the potential for recurrence years later, even without identifiable triggers. It underscores the need for high clinical suspicion in post-stroke patients with new neuromuscular deficits, the utility of serial electrophysiological evaluation for subtype classification, and the importance of timely immunotherapy and vigilant supportive care. Recognition of such atypical presentations is essential to reduce morbidity, prevent respiratory failure, and guide long-term follow-up for recurrent episodes.

## Introduction

Guillain-Barré syndrome (GBS) is an acute immune-mediated polyradiculoneuropathy characterized by rapidly progressive, symmetrical weakness, areflexia, and variable sensory or autonomic dysfunction, typically following an infection [1]. While generally monophasic, recurrent GBS (RGBS) is formally defined by the National Institute of Neurological Disorders and Stroke (NINDS) as two or more distinct episodes fulfilling diagnostic criteria, separated by at least two months after full recovery or four months after partial recovery, with occurrence in fewer than 6% of cases. Although infectious triggers are well recognized, GBS following cerebrovascular events is exceedingly rare, with only sporadic cases reported worldwide [2]. Post-stroke immune dysregulation, through mechanisms such as blood-brain barrier disruption, antigen exposure, and maladaptive immune priming, has been proposed as a potential trigger for aberrant peripheral autoimmunity [3]. We report a unique case of GBS occurring shortly after ischemic stroke with spontaneous recurrence four years later, underscoring the exceptional rarity of post-stroke GBS and the importance of ongoing vigilance in post-stroke patients presenting with new neuromuscular weakness.

## Case presentation

A 67-year-old right-handed man with a history of hypertension presented four years ago with the sudden onset of left-sided upper and lower extremity weakness and slurred speech, with no facial deviation. On initial neurologic examination, his Glasgow Coma Scale (GCS) was 15/15, and motor power was grade 3/5 in the left upper and lower extremities. A brain MRI at that time revealed a right basal ganglia ischemic infarct. On the fourth day of his intensive care unit (ICU) admission for post-stroke management, he developed rapidly progressive quadriparesis. Neurological reassessment showed motor power of 2/5 in all four extremities, accompanied by generalized areflexia.

A reinfarction or hemorrhagic transformation was initially suspected; however, an urgent non-contrast head CT demonstrated no new ischemic or hemorrhagic changes. Nerve conduction studies (NCS) revealed findings consistent with the acute motor-sensory axonal neuropathy (AMSAN) variant of Guillain-Barré syndrome (GBS). Cerebrospinal fluid (CSF) analysis showed elevated protein at 76 mg/dL (reference range: 15-45 mg/dL) with a mildly increased white blood cell (WBC) count of 12 cells/μL (reference range: 0-5 cells/μL), consistent with albuminocytologic dissociation. He was treated with a five-day course of intravenous immunoglobulin (IVIG) at a total dose of 2 g/kg over five days, leading to gradual motor recovery over the following weeks. He remained functionally independent and asymptomatic for the subsequent four years.

At present, four years following his initial presentation, the patient developed a second episode of acute, ascending weakness of one day's duration, initially involving the lower limbs and progressing to the upper limbs. In contrast to his initial episode, which occurred in close temporal association with an acute ischemic stroke, the patient’s recurrent ascending weakness developed spontaneously without any identifiable preceding infection, vaccination, or systemic illness. He also reported mild dysarthria and shortness of breath. On examination, he was afebrile (temperature 36.8°C), normotensive (blood pressure 128/76 mmHg), with a heart rate of 82 beats per minute, a respiratory rate of 18 breaths per minute, and an oxygen saturation of 97% on room air. Neurologic assessment revealed symmetric flaccid quadriparesis (grade 4/5 in upper limbs and 2/5 in lower limbs), areflexia, and preserved sensation. Cranial nerve examination was normal, and there were no meningeal signs.

Within 24 hours of admission, he developed shortness of breath and rapid breathing, with a respiratory rate of 38 breaths per minute, a single breath count of 10, and an oxygen saturation of 88% on 3 L/min via nasal cannula, indicating impending respiratory failure. He was intubated and mechanically ventilated. CSF analysis demonstrated clear fluid with <5 cells/mm³, protein of 65 mg/dL, and glucose of 85 mg/dL, consistent with GBS (Table 1).

**Table 1:** Relevant laboratory parameters during recurrent GBS episode GBS: Guillain-Barré Syndrome, CSF: Cerebrospinal Fluid, Na+: Sodium Ion, K+ : Potassium Ion

Laboratory Parameters	Results	Normal Value
CSF Analysis		
White Blood Cell	< 5/µL	0–5 /µL
Protien	65 mg/dL	15–45 mg/dL
Glucose	85 mg/dL	45–80 mg/dL
Metabolic Panel		
Creatinine	0.8 mg/dl	0.67 - 1.17 mg/dl
Urea	18.8 mg/dl	17- 43 mg/dl
Na+	136 mmol/l	136 - 145 mmol/l
K+	3.7 mmol/l	3.5 - 5.1 mmol/l
C-Reactive Protein	2.5 mg/L	< 5 mg/L
Serology		
Rapid HIV Diagnostic test	Non-reactive	Non-reactive
Cytomegalovirus IgM	Negative	Negative
Epstein–Barr Virus IgM	Negative	Negative
Mycoplasma pneumoniae IgM	Negative	Negative

**Figure 1:**
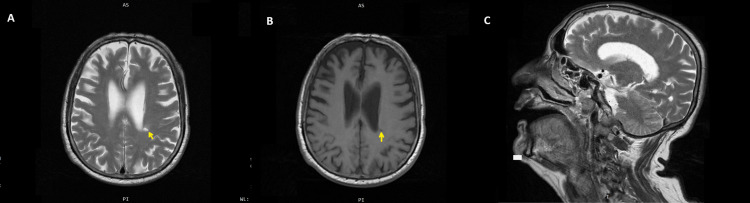
Brain MRI showing chronic lacunar infarct without evidence of acute infarction (A) T2-weighted imaging demonstrating an infarct in the left posterior ventricular area. (B) T1-weighted imaging depicting the corresponding infarct region. (C) Sagittal section illustrating normal structural anatomy for comparison. (Yellow arrow) Lacunar infarct

Brain MRI and MR angiography showed no evidence of acute infarction or vascular occlusion, with no acute DWI restriction, and revealed only chronic lacunar infarcts (Figure 1).

Repeat nerve conduction studies revealed prolonged distal motor latencies, reduced conduction velocities, conduction block, and absent F-waves in both upper and lower limbs, consistent with a symmetric sensorimotor demyelinating polyneuropathy. Reduced compound muscle action potential (CMAP) and sensory nerve action potential (SNAP) amplitudes indicated superimposed secondary axonal degeneration. These findings are consistent with AIDP. Serologic testing for cytomegalovirus (CMV), Epstein-Barr virus (EBV), mycoplasma, and HIV was negative, supporting the diagnosis of recurrent GBS without an identifiable infectious trigger.

A diagnosis of recurrent GBS was made. The patient underwent five sessions of plasmapheresis over 10 days (40 mL/kg per session). His clinical course was complicated by ventilator-associated pneumonia and urinary tract infection with Klebsiella pneumoniae identified on urine culture, both successfully treated with meropenem and vancomycin. He required temporary reintubation for respiratory distress but was subsequently weaned off ventilatory support and extubated successfully.

By the third week of admission, he demonstrated steady neurological improvement, with regained antigravity strength in all limbs and recovery of deep tendon reflexes. At discharge, he was ambulatory with mild residual lower-limb weakness (MRC grade 4+/5) and a Hughes GBS disability score of 2. He was referred for structured outpatient physiotherapy and continues to show improvement at follow-up.

## Discussion

Guillain-Barré syndrome (GBS) is an acute, immune-mediated polyradiculoneuropathy characterized by rapidly progressive, symmetrical weakness, areflexia, and variable sensory and autonomic dysfunction [1,4]. Although GBS typically follows antecedent infections, such as upper respiratory or gastrointestinal illness, its occurrence after cerebrovascular events is exceptionally rare. To date, only a few cases have been reported following hemorrhagic stroke, and a single case has been described after acute ischemic stroke [2]. In the present patient, the first episode of GBS developed during post-stroke intensive care, following an acute right basal ganglia ischemic infarct, a clinical scenario that initially prompted consideration of reinfarction or hemorrhagic transformation. Urgent neuroimaging ruled out recurrent cerebral ischemia, highlighting the diagnostic challenge of recognizing post-stroke GBS, which can mimic or complicate acute cerebrovascular disease.

Although the precise pathophysiology remains incompletely understood, stroke-induced immune dysregulation appears central. Ischemic brain injury triggers the release of damage-associated molecular patterns and pro-inflammatory cytokines into the systemic circulation via the compromised blood-brain barrier or CSF drainage pathways, promoting systemic immune activation and, paradoxically, transient immunosuppression [2,5]. Additionally, antigen-dependent autoimmunity may develop over time, with B-lymphocyte activation and CNS antibody production detected in a substantial proportion of stroke survivors, potentially predisposing them to peripheral nerve autoimmunity and subsequent GBS [3].

GBS is predominantly monophasic, with over 95% of patients experiencing a single episode; recurrence is rare, occurring in fewer than 6% of cases. Recurrent GBS (RGBS) is defined as two or more episodes fulfilling the National Institute of Neurological Disorders and Stroke (NINDS) diagnostic criteria, separated by at least two months if full recovery occurs or four months if partial recovery persists [6,7]. The patient described here developed a second, spontaneously occurring episode four years after the initial post-stroke GBS, without identifiable antecedent infection, vaccination, or systemic insult. This pattern emphasizes that recurrent episodes can arise independently of classical triggers, suggesting a role for underlying genetic susceptibility, dysregulated immune responses, or persistent autoantibody production [8].

Clinical manifestations in recurrent GBS often mirror prior episodes, though severity may vary. In this patient, both episodes were marked by rapid progression to quadriparesis, areflexia, and respiratory compromise, necessitating ventilatory support. Early recognition is critical, as delayed diagnosis can result in severe disability or mortality. Ancillary testing remains essential for confirming the diagnosis: cerebrospinal fluid analysis often demonstrates albuminocytologic dissociation, though early lumbar puncture may yield normal protein levels [9].

Nerve conduction studies (NCS) are essential for confirming the diagnosis of Guillain-Barré syndrome (GBS) and differentiating its electrophysiological subtypes, which have distinct pathophysiological mechanisms and prognostic implications [10,11]. The AIDP subtype is characterized electrophysiologically by prolonged distal motor latencies, reduced conduction velocities, temporal dispersion, and partial conduction block, indicating primary segmental demyelination and secondary axonal loss. Conversely, axonal variants, including acute motor axonal neuropathy (AMAN) and acute motor-sensory axonal neuropathy (AMSAN), are distinguished by markedly reduced compound muscle action potential (CMAP) amplitudes with normal or near-normal conduction velocities and absence of conduction block, signifying direct axonal injury at the nodes of Ranvier. In AMAN, sensory nerve action potentials (SNAPs) remain preserved, whereas in AMSAN, both motor and sensory fibers are affected, resulting in reduced or absent SNAPs. These patterns carry prognostic importance; axonal forms are generally associated with more severe initial weakness, slower recovery, and less complete functional restitution compared to acute inflammatory demyelinating polyneuropathy (AIDP) [11]. In the present case, NCS, classified using the Hadden criteria, demonstrated AMSAN during the initial post-stroke episode and AIDP during the recurrent episode, illustrating the phenotypic variability that can occur between episodes.

Management of recurrent GBS follows standard immunomodulatory approaches, including intravenous immunoglobulin (IVIG) and plasmapheresis [12,13]. In this case, IVIG facilitated recovery during the initial post-stroke episode, while plasmapheresis successfully managed the recurrent episode complicated by respiratory failure and secondary infections. Supportive care, particularly vigilant respiratory monitoring, remains pivotal, as patients may develop autonomic dysfunction with potentially fatal complications [14]. Functional recovery in recurrent episodes is often variable, influenced by the severity of axonal damage, immune response pattern, and timeliness of therapy. Early initiation of immunotherapy and structured multidisciplinary rehabilitation, including intensive physiotherapy and occupational therapy, has been shown to accelerate motor recovery, minimize long-term disability, and improve overall quality of life in survivors. Long-term follow-up remains essential to detect potential relapses, manage fatigue, and address residual neuropathic pain or weakness [15].

This case underscores several critical points for clinical practice. First, GBS can complicate acute ischemic stroke, necessitating high suspicion when new neurological deficits evolve beyond the expected post-stroke pattern. Second, recurrence can occur years after full recovery, sometimes without identifiable triggers, emphasizing the need for longitudinal vigilance. Third, multimodal investigation, including CSF analysis, nerve conduction studies, and serologic antibody testing, enables accurate subtype classification and guides therapy. Finally, prompt immunotherapy combined with supportive care, particularly for respiratory compromise, is essential to minimize morbidity and mortality. Recognition of post-stroke and recurrent GBS, though rare, is crucial, as early diagnosis and timely intervention can dramatically improve patient outcomes.

## Conclusions

This case illustrates an exceptionally rare overlap between ischemic stroke and GBS, followed by a spontaneous recurrence four years later with a distinct electrophysiological profile. It underscores the potential role of stroke-induced immune dysregulation as a trigger for peripheral nerve autoimmunity and highlights that recurrent GBS can occur independently of classical antecedent events such as infection or vaccination. Clinicians should maintain a high index of suspicion when new or atypical weakness develops after stroke, as GBS may mimic reinfarction or hemorrhagic transformation. Prompt recognition, supported by cerebrospinal fluid analysis and serial nerve conduction studies, is essential for accurate diagnosis and subtype differentiation. Early immunotherapy, either intravenous immunoglobulin or plasmapheresis, alongside vigilant respiratory monitoring and comprehensive rehabilitation, remains the cornerstone of management. This case reinforces that although GBS is typically monophasic, recurrence is possible even after prolonged remission, emphasizing the need for long-term follow-up and awareness of its variable clinical and electrophysiological manifestations to optimize outcomes.
